# Validation of the Assess Respiratory Risk in Surgical Patients in Catalonia (ARISCAT) Score for Predicting Postoperative Pulmonary Complications After Laparotomy in an Indian Population in the Present Era: A Retrospective Study

**DOI:** 10.7759/cureus.92743

**Published:** 2025-09-19

**Authors:** Rama Krishna Prasad Chikkala, A Chaitanya Pratyusha, Padmaja Durga, Pratyusha Patlolla, Supraja Ponduru, Metta Rajasekhar, Virinchi Sanapala

**Affiliations:** 1 Anaesthesiology and Critical Care, All India Institute of Medical Sciences (AIIMS) Bibinagar, Hyderabad, IND; 2 Anaesthesiology, Nizam’s Institute of Medical Sciences (NIMS), Hyderabad, IND; 3 Anaesthesiology, Employees State Insurance Corporation (ESIC) Medical College, Hyderabad, IND; 4 Anaesthesiology, All India Institute of Medical Sciences (AIIMS) Mangalagiri, Guntur, IND; 5 Anaesthesiology, Tata Memorial Centre, Mumbai, IND

**Keywords:** anesthesia, ariscat score, laparotomy, respiratory insufficiency, risk assessment

## Abstract

Introduction

Postoperative pulmonary complications (PPCs) lead to increased ICU admissions, duration of hospital stay, and mortality. Optimizing the modifiable risk factors before surgery helps reduce PPCs. A risk score named Assess Respiratory Risk in Surgical Patients in Catalonia (ARISCAT) was designed for the prediction of PPCs in the surgical group of patients by a study conducted in Europe. The study aims to test the generalizability of the ARISCAT risk score for PPCs in our population.

Methods

Data on preoperative risk factors and the occurrence of postoperative pulmonary complications, as defined in the ARISCAT study, were collected from the medical records of 539 patients undergoing surgery under general anesthesia during February 2017 to February 2020, and 501 were included in the final analysis. Categorical data was compared using the Chi-square test. Regression analysis was performed to assess the relationship between individual risk factors and the occurrence of PPC. The goodness of fit was tested using the Hosmer-Lemeshow test.

Results

Incidence of PPC did not show a statistically significant difference for age (p=0.855), preoperative respiratory infection (p=0.859), site of incision (p=0.523), duration of surgery (p=0.191), nature of surgery (emergency/elective) (p=0.922), preoperative anemia (p=0.388), albumin (p=0.393), and length of hospital stay (p=0.393). The overall incidence of PPC was 25 (4.9%). The receiver operating characteristic (ROC) curve showed an area under the curve (AUC) of 0.567 with a 95% CI of 0.5-0.7. The p-value for goodness of fit was 0.0001. The logistic regression analysis was insignificant.

Conclusion

The ARISCAT score was unable to discriminate between low- and high-risk groups for PPCs in our study population and may have limited utility in similar institutional settings.

## Introduction

Postoperative pulmonary complications (PPCs) have been found to have an association with long-term mortality [1]. PPCs also increase hospital stay and the need for intensive care unit (ICU) care [2]. Identifying and optimizing modifiable risk factors before surgery helps reduce PPCs. The Assess Respiratory Risk in Surgical Patients in Catalonia (ARISCAT) score [3], Predicting Post-operative Pulmonary Complications in Europe (PERISCOPE) study [4], Postoperative Pneumonia Risk Index [5], and Respiratory Failure Risk Index [6] have developed predictive models for PPCs.

A high-risk score, named Assess Respiratory Risk in Surgical Patients in Catalonia (ARISCAT), for PPC was proposed by a study conducted in Europe [3], which comprised 2,464 patients and recorded 252 events in 123 patients (5%). This study aimed to evaluate the validity and generalizability of the ARISCAT risk score in predicting postoperative pulmonary complications among adult patients undergoing open laparotomy under general anesthesia at a tertiary care center in India, with an emphasis on the high-risk group.

## Materials and methods

Methods

This is a retrospective, single-center study. The requirement for written informed consent was waived as this is a retrospective study based on data from clinical records. Nizam’s Institute of Medical Sciences (NIMS) Institutional Ethics Committee issued approval with number EC/NIMS/2849/2021, dated: 27-10-2021. A list of unique patient identification numbers was prepared from the operation theatre records to include all the patients who underwent surgery under general anesthesia in our hospital between February 2017 and February 2020, while excluding laparoscopic surgeries, superficial surgeries, and procedures related to previous postoperative complications. A total of 539 case records were collected from the hospital's medical records department using the prepared list. A team of 10 abstractors studied the case records over two months. Each case record was studied by two abstractors to avoid errors. Data relevant to the study were collected manually in standardized data forms. Abstractors' bias was overcome by keeping them blinded to the study's aims and objectives and involving them only in data collection. All the abstractors were trained in data collection and underwent a pilot test before the study commenced. The abstractors were monitored regularly by randomly cross-checking some of the case records with the data collected to ensure quality.

Data collection

Data extracted from the case records focused on i) Demographic data, which included age, gender, BMI, diagnosis, the planned surgery, and American Society of Anesthesiologists (ASA) class. ii) Variables of the seven risk parameters of the ARISCAT score [3], namely age, preoperative oxygen saturation, respiratory infection in the last month, hemoglobin, site of surgical incision, duration of surgery, and nature of surgery, whether elective or emergency. iii) Postoperative pulmonary complications as defined in the ARISCAT study [3], namely respiratory failure (PaO_2_<60 on room air or PaO_2_/FiO_2_<300 or SpO_2_ <90 or need for O_2_ therapy), suspected pulmonary infection (new or changed sputum/new or changed lung opacities in X-ray/temperature >38°C/leucocyte >12,000/mm^3^), pleural effusion (blunting of costophrenic angles or loss of silhouette of ipsilateral hemidiaphragm in upright posture), atelectasis (lung opacification with a shift of mediastinum, hilum, and hemidiaphragm toward the affected area or compensatory overinflation of nonatelectatic lung), aspiration pneumonitis (respiratory failure after inhalation of gastric contents), bronchospasm (new expiratory wheeze responding to bronchodilators), and pneumothorax (air in pleural space with no vascular bed surrounding the visceral pleura) during the first five postoperative days. PPC was defined as the occurrence of at least one event on a list of in-hospital fatal or nonfatal PPCs. iv) Additional parameters to include preoperative albumin levels, postoperative length of hospital stay, and postoperative mechanical ventilation were also extracted.

Exclusion of case records

Sixteen case records of procedures related to previous postoperative complications, pregnancy, and patients with preoperative intubated trachea or tracheostomy were excluded. Twenty were excluded due to incomplete preoperative data. Five hundred three case records were then studied for the occurrence of PPCs. Five hundred one case records were included in the final analysis after excluding two cases where death occurred due to reasons not related to PPC.

Statistical analysis was performed using the Statistical Package for the Social Sciences (SPSS) version 21 (SPSS Inc, IBM, Chicago, IL, USA). The required sample size for the study has come to 457, assuming a 5% incidence of PPC according to the ARISCAT study [3], with a 95% confidence interval and 2% absolute precision with the formula n = (Z^2 * p * (1-p))/E^2, where Z is the Z-statistic for the desired confidence level, p is the estimated proportion, and E is the desired margin of error (absolute precision). Data represented as frequency and percentage. Categorical data was compared using the Chi-square test. A two-sided p-value of <0.05 was considered significant. Binary regression analysis was performed to assess the relationship between individual risk factors and the occurrence of PPC and represented as an odds ratio. The goodness of fit was tested using the Hosmer-Lemeshow test.

## Results

Out of 539 case records, 36 were excluded based on exclusion criteria, two were not analyzed due to death unrelated to PPCs, and 501 case records were analyzed. Table 1 gives the incidence of postoperative pulmonary complications in our cohort. Only two (0.4%) patients were over 80 years (high-risk age group), and none developed PPCs. In contrast, 340 (67.9%) were under 50 years (low risk). The incidence of PPC did not show a statistically significant difference in the age category (p=0.855). Preoperative respiratory infections were presented in 74 (14.8%) of our patients compared to 5.7% in the ARISCAT cohort. However, only four (5.4%) of these patients developed PPCs, and the association was not statistically significant.

**Table 1:** Incidence of postoperative pulmonary complications as a percentage in each category of ARISCAT risk score parameters Data expressed in frequency (n) and percentage of patients (%). A p-value less than 0.05 is considered significant. Chi-square test applied. PPC: postoperative pulmonary complications, n: number of patients, ARISCAT: Assess Respiratory Risk in Surgical Patients in Catalonia.

S. no	Parameter	Category	Total frequency (n=501)	PPC (n=25)	No PPC (n=476)	Chi-square value	p-value
1	Age(years) n (%)	<50	340 (67.9%)	16 (4.7%)	324 (95.3%)	0.314	0.855
		51-80	159 (31.7%)	9 (5.6%)	150 (94.4%)		
		≥81	2 (0.4%)	0	2 (100%)		
2	Preoperative SpO_2_	0	496 (99%)	25 (4.9%)	471 (94.01%)	0.265	0.876
		8	3 (0.59%)	0	3 (0.59%)		
		24	2 (0.4%)	0	2 (0.4%)		
2	Incision n (%)	Upper abdominal	452 (90.2%)	22 (4.86%)	430 (95.14%)	1.29	0.523
		Peripheral	42 (8.4%)	2 (4.76%)	40 (95.2%)		
		Intrathoracic	7 (1.4%)	1 (14.2%)	6 (85.8%)		
3	Nature of surgery n (%)	Elective	479 (95.6%)	24 (5%)	455 (95%)	0.095	0.922
		Emergency	22 (4.4%)	1 (4.5%)	21 (95.5%)		
4	Hemoglobin (g/dL) n (%)	≥10	340 (67.9%)	17 (5%)	323 (95%)	0.746	0.388
		<10	161 (32.1%)	8 (4.96%)	153 (95.04%)		
5	Respiratory infection n (%)	Yes	74 (14.8%)	4 (5.4%)	70 (94.6%)	0.03	0.859
		No	427 (85.2%)	21 (4.9%)	406 (95.1%)		
6	Surgical duration n (%)	<2	51 (10.2%)	2 (3.9%)	49 (96.1%)	3.31	0.191
		2-3	51 (10.2%)	0	51 (100%)		
		>3	399 (79.6%)	23 (5.7%)	376 (95.3%)		
7	ARISCAT risk grade	Low (<26)	40 (7.98%)	0	40 (100%)	5.08	0.079
		Intermediate (26-44)	320 (63.8%)	14 (4.3%)	306 (95.7%)		
		High (≥45)	141 (28.1%)	11 (7.8%)	130 (92.2%)		

Anemia was present in 161 (32.1%) patients in our population, much higher than the 6.5% reported in the ARISCAT study. However, only eight (4.9%) of these patients developed PPCs, showing no statistically significant association. Emergency surgeries were performed in 22 (4.4%) patients, lower than the ARISCAT rate of 14.3%, and none developed PPCs. Upper abdominal surgeries made up 452 (90.2%) of the procedures, with a PPC incidence of 22 (4.86%) cases. Peripheral surgeries accounted for 42 (8.4%) with two (4.7%) PPC incidence, while seven (1.4%) were intrathoracic with one (14.2%) PPC reported. There was no statistical significance with the type of incision as a risk factor for PPC in our study population.

A higher proportion of our patients, 399 (79.6%), had prolonged surgeries (>3 hours) compared to 9.4% in the ARISCAT cohort, yet only 23 (5.7%) developed PPCs. Duration of surgery was not a statistically significant predictor of PPC in our population. The mean surgery duration for patients with PPCs was 6.22 hours, compared to 5.52 hours for those without, this difference was not statistically significant. The average hospital stay in our study was eight days, longer than the three days reported in the ARISCAT study. Patients with PPCs had a mean stay of 7.22 days versus 8.95 days for those without PPCs, with no significant difference (p=0.39).

The mean serum albumin levels were 3.22 g/dL in patients who developed PPCs and 3.44 g/dL in those who did not, with no statistically significant difference (p=0.39), indicating albumin was not a significant risk factor. The overall incidence of PPCs in our study was 25 (4.9%). Postoperative ventilation was required in 57 (11.2%) patients, with 25 (4.9%) needing ventilation for more than 12 hours.

**Figure 1:**
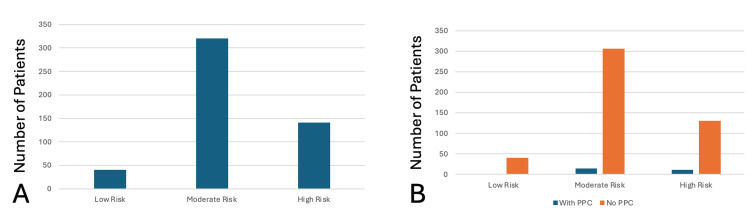
Incidence of postoperative pulmonary complications (PPCs) in different risk classes A: A bar diagram depicting the number of patients belonging to low risk, moderate risk, and high risk. B: A bar diagram depicting the incidence of PPC in different risk classes.

Based on ARISCAT score stratification, 40 (8%) patients were classified as low risk, 320 (64%) as moderate risk, and 141 (28%) as high risk (Figure 1A). PPCs occurred in 11 (7.8%) high-risk patients, accounting for 44% of total PPCs, and in 14 (4.3%) moderate-risk patients, accounting for 56%. No PPCs were observed in the low-risk group (Figure 1B).

**Figure 2:**
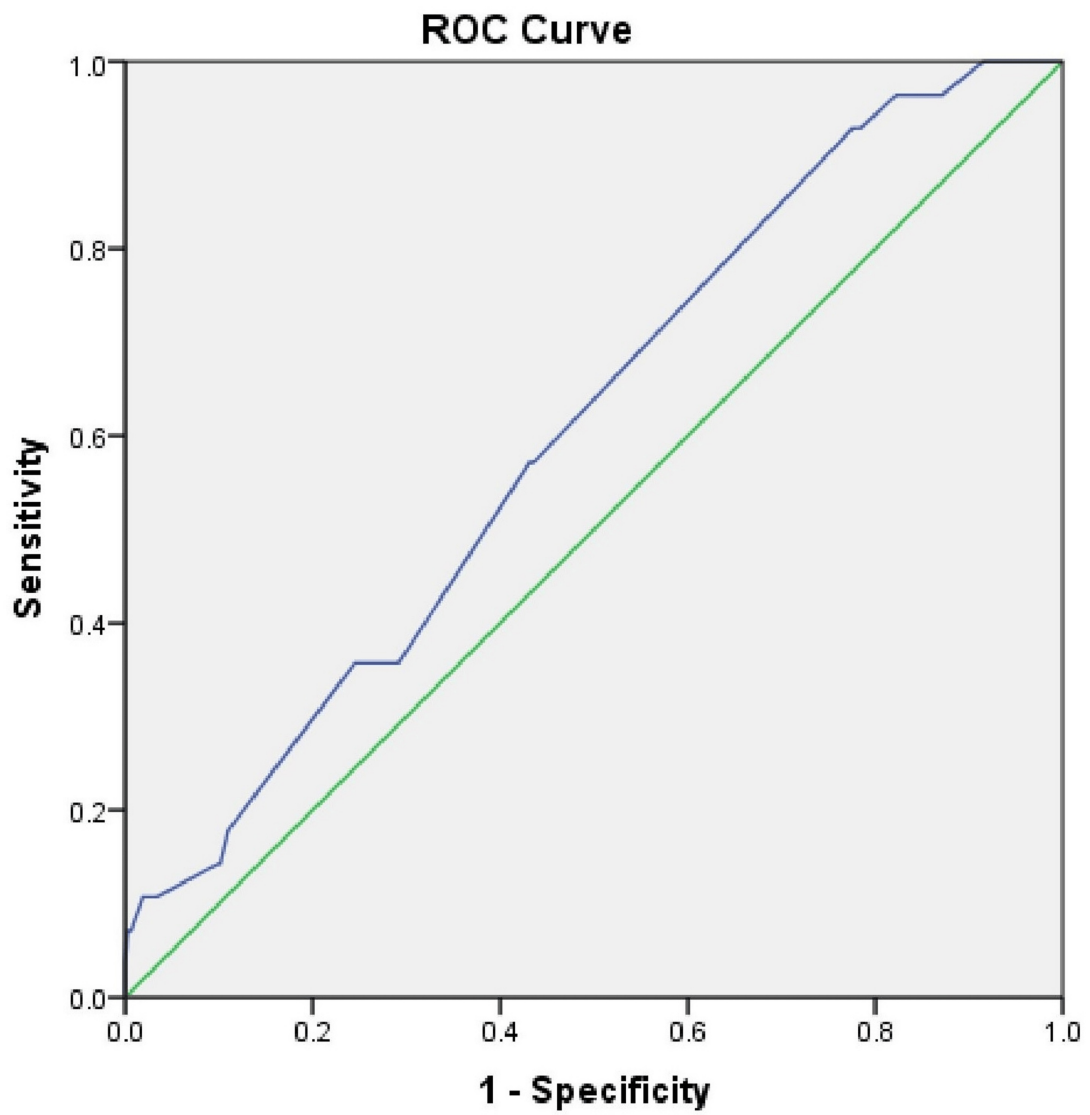
ROC curve for ARISCAT score for PPCs The incidence of PPC in our study population has given an AUC of 0.567 with 95% CI 0.5-0.7. ROC: receiver operating characteristic, ARISCAT: Assess Respiratory Risk in Surgical Patients in Catalonia, PPC: postoperative pulmonary complication, AUC: area under the curve.

Postoperative ventilation was required in 22 (15.6%) high-risk patients (37.5% of total ventilated cases), 32 (9.9%) moderate-risk patients (57.2%), and three (7.5%) low-risk patients (5.3%). The receiver operating characteristic (ROC) curve for the ARISCAT score for the incidence of PPC in our study population has given an area under the curve (AUC) of 0.567 with 95% CI 0.5-0.7; p< 0.05 (Figure 2).

Binary logistic regression evaluation of ARISCAT risk parameters for predicting postoperative pulmonary complications was insignificant (Table 2). The p-value for goodness of fit for the high-risk category according to the ARISCAT score for PPCs was 0.0001 when the Hosmer-Lemeshow test was used.

**Table 2:** Logistic regression modeling of ARISCAT score predictors for postoperative pulmonary complications A p-value less than 0.05 is considered significant. SpO_2_: saturation, ARISCAT: Assess Respiratory Risk in Surgical Patients in Catalonia.

S. no	ARISCAT risk parameter	Odds ratio (OR)	p-value
1	Age	0.99	0.951
2	Preoperative SpO_2_	0.184	0.99
3	Respiratory infection in the last month	0.99	0.807
4	Preoperative anemia	1.007	0.855
5	Duration of surgery	1.02	0.451
6	Surgical incision	1.029	0.596
7	Nature of operation (emergency)	1.027	0.839

## Discussion

Compared to the ARISCAT study, our population had a higher proportion of high-risk patients, yet the incidence of PPCs was similar (4.9% vs. 4.4%). Notably, high-risk patients did not show a significantly higher PPC incidence in our study. Age and incision site are the non-modifiable risk factors for PPCs. Effective thoracic epidural analgesia may have reduced respiratory compromise after upper abdominal surgery, contributing to the low PPC rate. Routine pre- and postoperative inspiratory muscle training in our patients may have reduced PPCs, as incentive spirometry and chest physiotherapy are known to improve postoperative respiratory function [7-10].

Despite a high prevalence of preoperative respiratory infections or chronic respiratory disease, PPC incidence was low. This may be due to preoperative optimization with antibiotics and continued bronchodilator therapy in patients with obstructive airway disease [11]. Preoperative chest physiotherapy and postoperative early mobilization, both proven to reduce PPCs [12], are routinely practiced in our hospital. Hence, preoperative respiratory infection may be considered a modifiable risk factor, aligning with findings by Kim et al. [13] in mild to moderate chronic obstructive pulmonary disease (COPD).

Although emergency surgery is a known risk factor for PPC [14-15], no PPCs occurred in such cases in our study, possibly due to standardized postoperative respiratory support protocols applied to all patients. The low PPC incidence in our study may be attributed to comprehensive preoperative optimization, smoking cessation, hemoglobin and albumin correction, respiratory therapies, and standardized postoperative care, including epidural analgesia, early ambulation, deep vein thrombosis (DVT) prophylaxis, physiotherapy, and balanced nutrition.

A prospective study by Warner et al. involving 200 patients showed reduced PPC risk with smoking cessation over two months preoperatively [16]. Our institutional protocol enforces strict adherence to preoperative smoking cessation. Hypoalbuminemia, a known PPC risk factor per the Respiratory Failure Risk Index by Arozullah et al. [17], is addressed in our institute through preoperative nutritional optimization and albumin supplementation when needed.

An AUC of 0.567 indicates poor discriminatory performance of the ARISCAT score in our population. The goodness-of-fit test (p=0.0001) further suggests that high-risk categorization did not predict PPCs effectively. Implementation of Enhanced Recovery After Surgery (ERAS) protocols across all perioperative phases may have contributed to the reduced PPC incidence despite a high-risk population. The ARISCAT study included diverse healthcare settings, while our study was limited to a single tertiary center, possibly explaining the lower PPC incidence. Prospective multicenter studies are needed to validate risk scores and help reduce PPC rates.

## Conclusions

The ARISCAT score for prediction of risk of postoperative pulmonary complications could not discriminate between low- and high-risk groups in our study population and may have limited utility in similar institutional settings. There is a need to develop new risk stratification scoring systems based on the present clinical practices and advances in the medical field. A comprehensive preoperative optimization and standardized postoperative care may act as important factors to reduce PPC, even in the high-risk ARISCAT group.
